# Collecting rare diseases

**DOI:** 10.12688/f1000research.5577.1

**Published:** 2014-10-31

**Authors:** Sean Ekins

**Affiliations:** 1 Collaborations in Chemistry, Fuquay Varina, NC, 27526, USA; 2 Collaborative Drug Discovery, Burlingame, CA, 94010, USA; 3 Phoenix Nest, Brooklyn, NY, 11215, USA; 4 Hereditary Neuropathy Foundation, New York, NY, 10016, USA; 5 Hannah's Hope Fund, Rexford, NY, 12148, USA

## Abstract

This editorial introduces the *F1000Research* rare disease collection. It is common knowledge that for new treatments to be successful there has to be a partnership between the many interested parties such as the patient, advocate, disease foundations, the academic scientists, venture funding organizations, biotech companies, pharmaceutical companies, NIH, and the FDA. Our intention is to provide a forum for discussion and dissemination of any rare disease related topics that will advance scientific understanding and progress to treatments.

## Editorial

**Figure 1.**
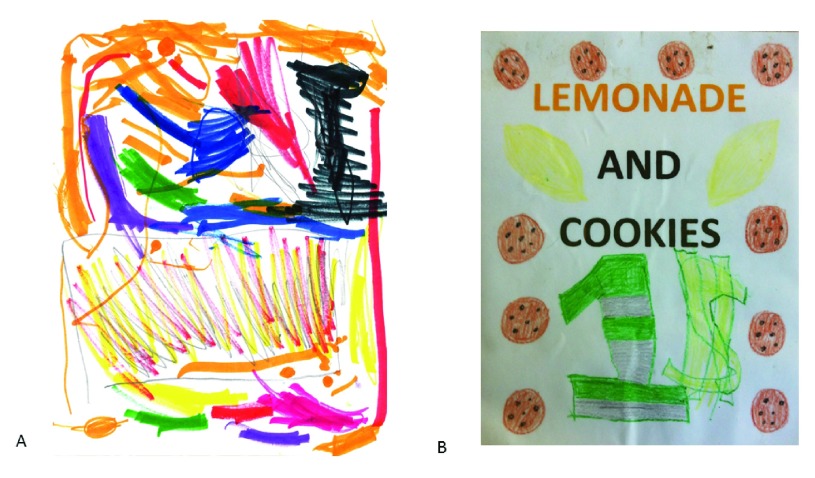
Images from rare disease pediatric patients **A**. courtesy of Jonah Weishaar ( http://jonahsjustbegun.org/) and **B**. courtesy of Hannah Sames ( http://www.hannahshopefund.org/). Both images have been used in fund raising activities.

Starting a collection of anything is always a challenge whether it is music, books, stamps or art etc., especially figuring out where you begin with something that’s seemingly infinite. With rare diseases we know that there are close to a near mythical numbering of 7000 or so. Do you start with all the diseases that belong to the Rare Diseases Clinical Research Network (RDCRN, https://www.rarediseasesnetwork.org/) or do you focus on one group such as the lysosomal storage diseases or the inherited neuropathies as examples? Or do you look elsewhere for a good starting point? Where does one even look if you want to learn about all of the rare diseases? Does one go to the NORD ( https://www.rarediseases.org/), Eurodis ( http://www.eurordis.org/), Orphanet ( http://www.orpha.net/consor/cgi-bin/index.php), Wikipedia ( http://en.wikipedia.org/wiki/Rare_disease) websites etc.? There are few specific rare disease related journals like Orphanet Journal of Rare Diseases. Do you start from A and work through to Z? Another approach could be purely random, address them as the rare disease cards are dealt. If you are a parent or a patient, you do not have a choice, you have to learn about the disease that afflicts you or your child. You have to assimilate the science, the scientists and in most cases the unknown. The fact that there may not be a treatment or even one on the horizon is not one that many can contemplate but for those that do, this is just one of many challenges that lie ahead as well as creative ways to raise funds ( Figure 1).

Much has been written about rare diseases and the hunt for treatments and the issues that surround them ^1– 8^. They do not discriminate with respect to rich or poor, geographic location, but what is still striking is the efforts that it takes try to find treatments if you are a patient or parent. Do not assume a pharmaceutical company, biotech or academic researcher is working on your disease. The rare disease odyssey has been summarized previously ^9^ encompassing everything from fund raising to starting foundations and companies. But more parents and patients are taking on the rare diseases head on to diagnose the disease ^10^ as well as try to find or fund a treatment.

When I was invited to edit this collection it was suggested that we try to cover disease awareness, drug research, development, policies, clinical studies, etc. and to disseminate this knowledge through the *F1000Research* open access platform. While that goes some way towards what we are trying to achieve, I think we also need to raise awareness of what can be done if you are a parent or patient too. One of the unwritten challenges a (non-scientist) rare disease patient or parent still faces is the difficulty in publishing a paper on their own. Having previously helped rare disease parents/patients to get their ideas in papers ^9^ and grants as a co-author I have seen their insights, vision and been stunned by what they continue to do and face. I have seen many of them respected as peers in the scientific community and yet others are shunned. But it’s rarer for them to be allowed to publish their thoughts alone in a peer reviewed journal. I find this bemusing, they have already overcome incredible challenges, diagnosis, fund raising, finding scientists, and then at the final hurdle they hit a wall in publishing their findings. They can appear at rare disease conferences as speakers and panelists, they can be the subjects of popular articles in magazines or on television documentaries. Their life story can be documented in a movie, they can blog, and use Facebook or Twitter to reach out, but publishing a peer reviewed paper is a whole new challenge. But it is also crucial if the majority of scientists or others in the field are to see their ideas and they in turn are to be credited for them. Still, in this day and age the scientific paper is the currency to get funding and be acknowledged as an expert, and perhaps this is even more important in the rare disease community. One would expect to see more collaboration and sharing, but that does not seem to be the case, perhaps for these reasons, which all come down to the shortage of money. The US National Institute of Health (NIH) and other funding bodies are still focused predominantly on funding academic scientists, but these parents and patients are the font of knowledge on the diseases they represent. They would make terrific PI’s because of their no-nonsense attitude and urgency. Perhaps in some small way this collection can rectify this situation and while many of the articles will be from scientists, I hope we can also provide an outlet for the rare disease patient and parent voice, which deserves to be heard. Many of them have made significant contributions to medicine which because of them not being PhD’s or affiliated with scientific institutions, leads to discrimination. Good ideas can come from anywhere, and the rare disease community members I hope will be inspired to keep having them.

Interest in rare diseases will continue to grow and more scientists will be recruited to the field. Having the option for publications that are open goes a long way to starting collaborations and sharing knowledge more readily. Can we show that rare diseases are not only profitable but also that the ‘process of rare disease drug discovery and development’ itself can be considerably more efficient? Then we might be able to industrialize the process and make some headway at collecting a few more rare diseases as those with approved treatments.
